# Infertility in a man with oligoasthenozoospermia associated with mosaic chromosome 22q11 deletion

**DOI:** 10.1002/mgg3.487

**Published:** 2018-11-20

**Authors:** Yanyan Liu, Hongmei Zhu, Xuan Zhang, Ting Hu, Zhu Zhang, Jing Wang, Yi Lai, Jiemei Zheng, Dan Xie, Bei Xia, Li Qin, Liangyu Xie, Shanling Liu, He Wang, Huaqin Sun

**Affiliations:** ^1^ Prenatal Diagnosis Center, Department of Obstetrics & Gynecologic Key Laboratory of Birth Defects and Related Diseases of Women and Children (Sichuan University) Ministry of Education West China Second University Hospital, Sichuan University Chengdu China; ^2^ SCU‐CUHK Joint Laboratory for Reproductive Medicine Key Laboratory of Birth Defects and Related Diseases of Women and Children (Sichuan University) Ministry of Education Department of Pediatrics West China Second University Hospital, Sichuan University Chengdu China

**Keywords:** 22q11 deletion, gonosomal mosaic, karyotypes, oligoasthenozoospermia

## Abstract

**Background:**

A 30‐year‐old oligoasthenozoospermia man was found to have unbalance mosaic translocation between chromosome 22 and four other chromosomes (5, 6, 13, and 15) during the investigations for a couple with infertility for 3 years, which is a rare event in human pathology.

**Methods:**

Classical cytogenetics analysis, fluorescence in situ hybridization (FISH), and chromosome microarray analyses (CMA) were performed on peripheral blood lymphocytes; copy number variation sequencing (CNV‐Seq) analysis was performed on sperm DNA.

**Results:**

Classical cytogenetics analysis showed the presence of six cell lines on peripheral blood lymphocytes: 45, XY, der (13) t(13;22),‐22[10]/46, XY, t(13;22)[6]/45, XY, der(15)t(15;22),‐22[4]/46, XY, t(13;22)[1]/45, XY, der(5)t(5;22),‐22[1]/45, XY, der(6)t(6;22)[1]. FISH and CMA performed on peripheral blood cells showed the presence of a 6.9 Mb mosaic 22q11 deletion (approximately 50% of cells); it is unexpected that the phenotypes of this man were merely oligoasthenozoospermia, mild bradycardia, and mild tricuspid regurgitation. CNV‐Seq analysis performed on sperm DNA revealed the rate of 22q11 deletion cells was obviously lower compared with peripheral blood cells. And the frequency of gametes exhibiting a normal or balance chromosomal equipment was above 80% in sperm samples.

**Conclusion:**

To the best of our knowledge, this report is the first case of a de novo gonosomal mosaic of chromosome 22q11 deletion just associated with male infertility.

## 1 INTRODUCTION

Infertility is a major health problem, which affects approximately 22% of married couples in reproductive age. Chromosomal defects are the most common genetic abnormalities in infertile men, with an incidence of cytogenetic abnormalities ranging from 2.1% to 15.5% (Chandley et al., 1975).

The 22q11 region is susceptible to chromosomal rearrangements leading to DiGeorge syndrome (DGS/VCFS), cat eye syndrome (CES), and 22q11.2 distal deletion syndrome (Edelmann, Pandita, & Morrow, 1999; McDermid & Morrow, 2002). 22q11 deletion syndrome (22q11DS), also known as DiGeorge syndrome or velocardiofacial syndrome, is the most common chromosomal microdeletion disease, and this disease showed a variety of phenotypes, such as velopharyngeal insufficiency, facial anomalies, cardiac defects, immunodeficiency, and mental retardation (Cohen, Chow, Weksberg, & Bassett, 1999; Emanuel, 2008). 22q11.2 distal deletion syndrome showed abnormality of the face, intellectual disability, short stature, and small for gestational age.

In biological systems, mosaicism implies the presence of more than one genetically distinct cell line in a single organism. Mosaicism can be caused by DNA mutations, epigenetic alterations of DNA, chromosomal abnormalities, and the spontaneous reversion of inherited mutations (Youssoufian & Pyeritz, 2002). The pattern of mosaic distribution of mutations is largely determined by normal embryological processes of cell replication, cell migration, and apoptosis, and by the timing and pathophysiological effects of the mutation (Biesecker & Spinner, 2013; Youssoufian & Pyeritz, 2002). There are three specific types of mosaicism that describe which parts of the body harbor the variant cells and the potential for transmission to offspring; these include germ line mosaicism, somatic mosaicism, and gonosomal mosaicism (Biesecker & Spinner, 2013; Youssoufian & Pyeritz, 2002).

In the present study, we firstly reported a gonosomal mosaic of chromosome 22q11 deletion found in an infertility male without any severe clinical manifestations except for oligoasthenozoospermia, mild bradycardia, and mild tricuspid regurgitation.

## 2 PATIENT AND METHODS

### 2.1 The patient

The patient, a 30‐year‐old male, the only son of healthy consanguineous parents, was referred to our center for infertility investigations. His physical examination was normal, and the family history was unremarkable; the school attendance and intelligence of this patient were normal; molecular analysis of microdeletions (AZF regions and SRY gene) disclosed that no Y chromosome microdeletions existed; the levels of FSH, LH, free T, and PRL were within normal limits. Two different semen analyses of patient were carried out according to the World Health Organization criteria (1999), and the results showed severe oligoasthenozoospermia: Sperm volume was only 1.5 ml, sperm concentration was only 0.8 million/ml, and sperm progressive motility (A + B quality) was 15.7%. Electrocardiogram showed that his heart rate was 58 beats per minute, and ultrasonic cardiogram showed that he had mild tricuspid regurgitation.This study was approved by the ethics board at West China Second University Hospital, Sichuan University, China. Informed consent of the legal representatives of this patient was obtained.

### 2.2 Cytogenetic analysis and FISH analyses

GTG‐banded chromosomes from cultured peripheral blood lymphocytes were tested. The two lymphocytes samples were obtained at different time with more than 6 months interval. Briefly, whole blood was cultivated for 72 hr at 37°C in lymphocyte culture medium. Twenty‐four metaphases were analyzed, and the karyotype results were described according to the International System for Human Cytogenetic Nomenclature (ISCN, 2016). FISH was performed on peripheral blood lymphocytes using the 22q11.2 probe (GP Medical, Beijing, China) and 13q14 probe as control.

### 2.3 CMA analysis and CNV‐seq analysis

We performed CMA analysis of DNA extracted from peripheral blood of this patient. Microarray testing was undertaken using the Affymetrix CytoScan 750K array. Data were analyzed using Chromosome Analysis Suite Software with copy number changes reported at a resolution of 200 kb.

Sperm DNA of this patient was analyzed by CNV‐seq, gDNA (50 ng) was fragmented, and DNA libraries were subjected to massively parallel sequencing using the NextSeq CN500 Platform (Illumina) to generate 5 million raw sequencing reads with 36‐bp genomic DNA sequences. Chromosome profiles were finally plotted as copy number (*Y*‐axis) versus chromosome pos (*X*‐axis); a blue line was used to indicate the mean copy number across each chromosome to identify the nature and map position of any deleted regions.

Identified and mapped CNVs were interrogated using publicly availed DECIPHER databases (https://decipher.sanger.ac.uk/).

## 3 RESULTS

**Figure 1:**
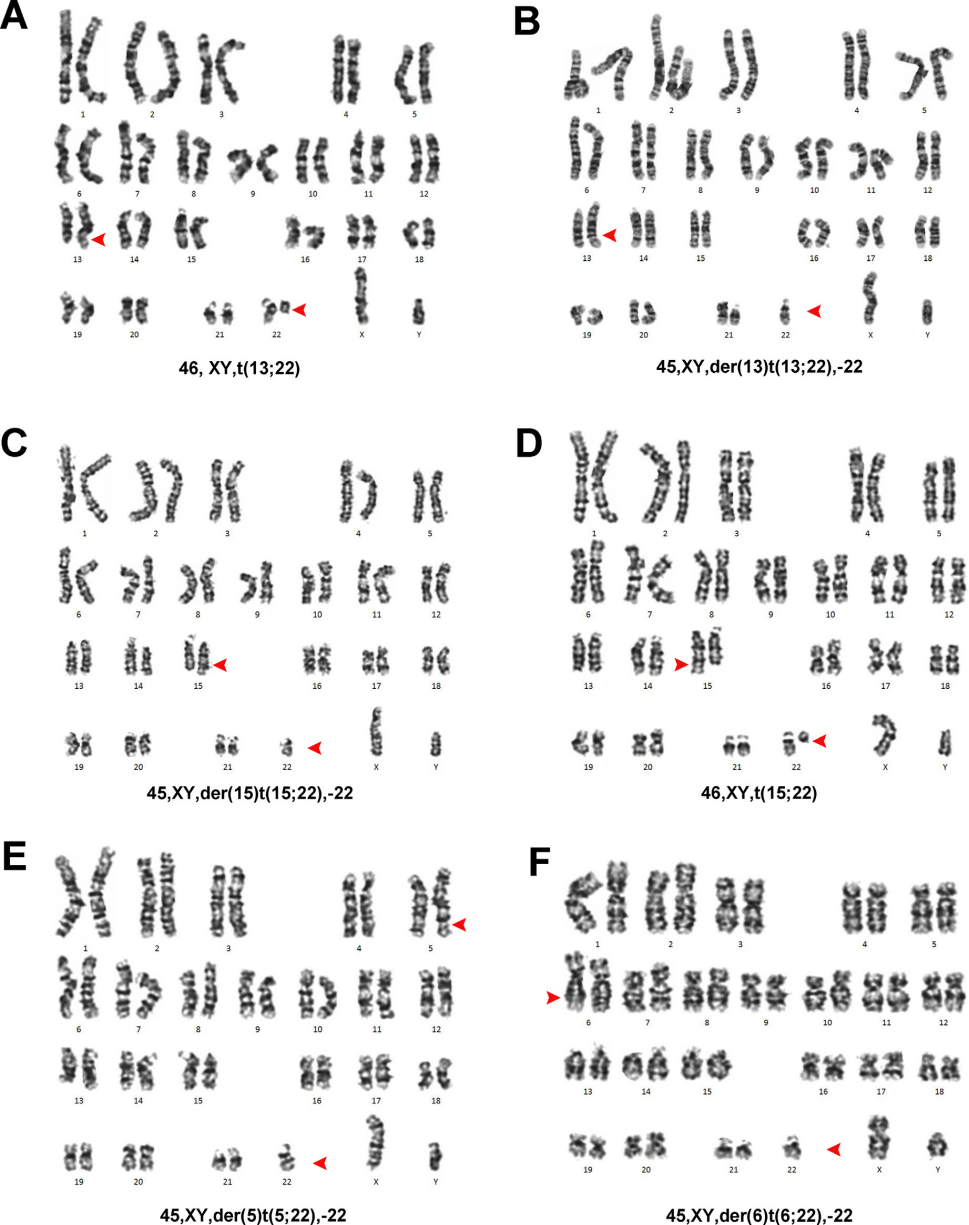
Six kinds of peripheral blood lymphocytes karyotypes of the patient. Arrowheads show chromosomal defects

The results of classical cytogenetics performed on peripheral blood lymphocytes revealed that 100% of the metaphase chromosome was abnormal, which showed the presence of six cell lines: 45, XY, der(13)t(13;22),‐22[10]/46, XY, t(13;22)[6]/45, XY, der(15)t(15;22),‐22[4]/46, XY, t(13;22)[1]/45, XY, der(5)t(5;22),‐22[1]/45, XY, der(6)t(6;22)[1]/45, XY, der(13)t(13;22),‐22[1] (Figure 1).

**Figure 2:**
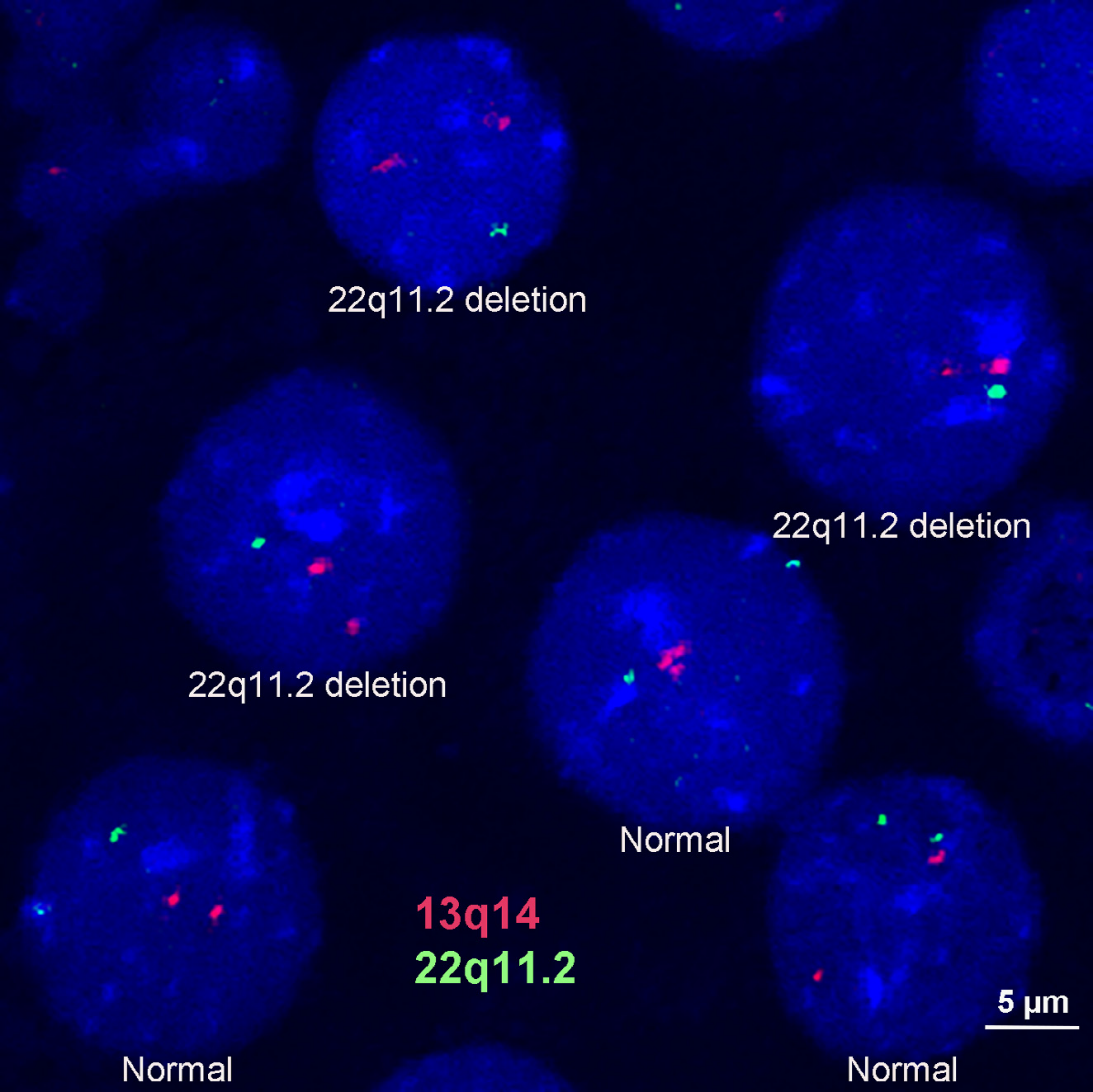
Fluorescence in situ hybridization (FISH) analysis on interphase chromosomes of the patient. FISH image of the patient showing two hybridization signals on 13q14 region (red) but mosaic of one hybridization signal on 22q11.2 region (green). Scale bar 5 μm

Moreover, a total of 100 nuclei were examined by FISH assay. Results demonstrated that all 100 nuclei showed two signals of the control probe at 13q14; however, approximately 50 nuclei (50%) showed only one signal for 22q11.2 probe (Figure 2). These results indicated a mosaic deletion of 22q11.2

**Figure 3:**
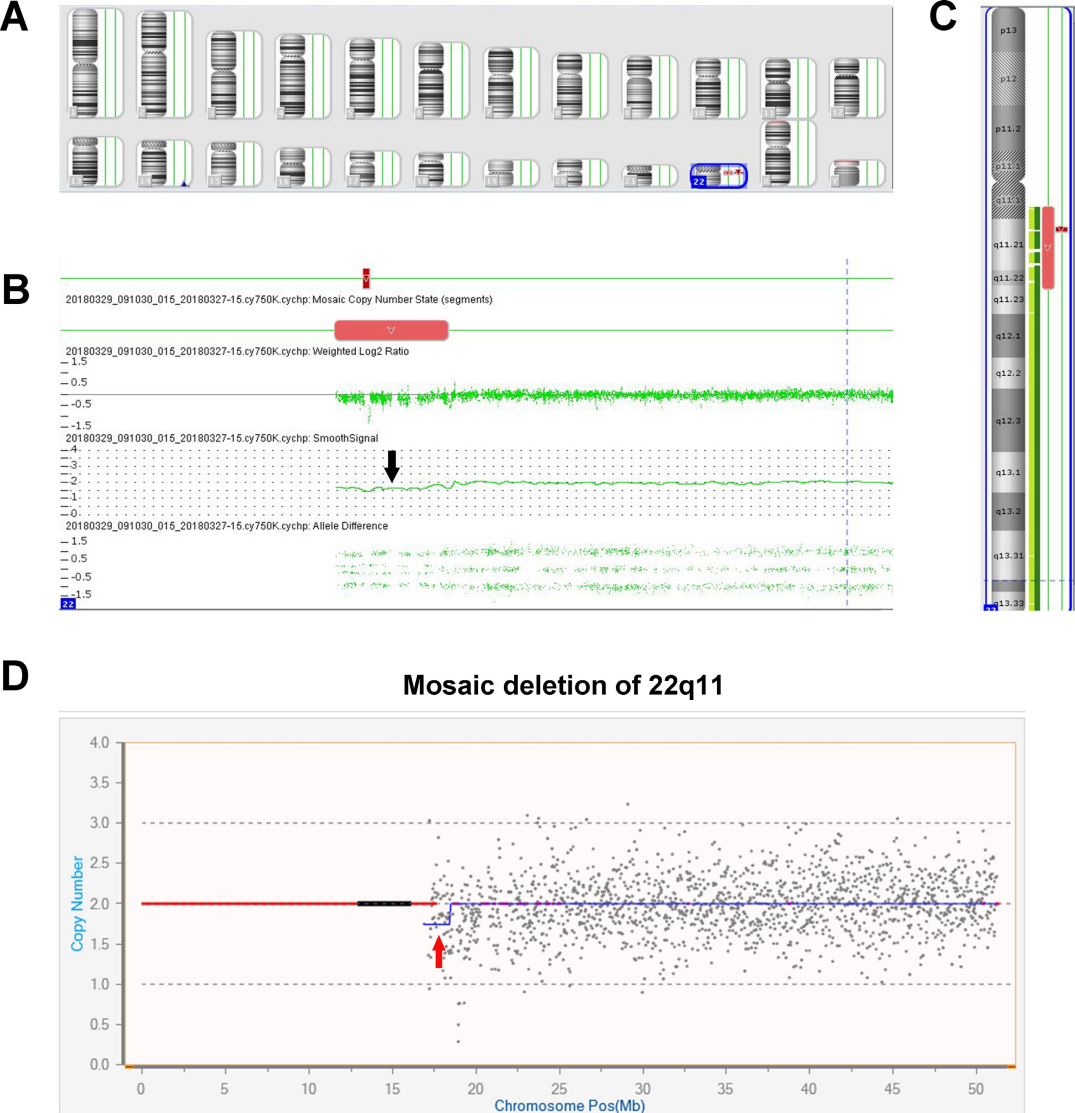
Chromosome microarray analyses (CMA) analysis on peripheral blood lymphocytes and copy number variation sequencing (CNV‐seq) profiles of sperm. The CMA analysis on peripheral blood lymphocytes (Panel a–c) shows a 6.9M mosaic deletion at chromosome 22q11 (approximately 50% of cells) marked by arrow in Panel b. The CNV‐seq profiles of sperm (Panel d) show low ratio of mosaic of chromosome 22q11 deletion marked by arrow

The chromosomal profiles of peripheral blood lymphocytes, obtained by CMA, showed the presence of a 6.9 Mb (chromosome 22:16,888, 899‐23,855,471) mosaic 22q11 deletion (approximately 50% of cells) (Figure 3a–c).

Copy number variation sequencing (CNV‐Seq) analysis performed on sperm DNA revealed the rate of 22q11 deletion cells was obviously lower compared with peripheral blood cells (Figure 3d). And the result showed that the frequency of gametes exhibiting a normal or balance chromosomal equipment was above 80% in sperm samples.

## 4 DISCUSSION

In the present study, we found an oligoasthenozoospermia male with complex chromosomal abnormality in peripheral blood cells. The two lymphocytes samples were obtained at different time with more than 6‐month interval, and classical cytogenetics analysis showed the presence of six cell lines in peripheral blood: 45, XY, der (13) t(13;22),‐22[10]/46, XY, t(13;22)[6]/45, XY, der(15)t(15;22),‐22[4]/46, XY, t(15;22)[1]/45, XY, der(5)t(5;22),‐22[1]/45, XY, der(6)t(6;22), ‐22[1]/45, XY, der(13)t(13;22),‐22[1]. FISH and CMA showed the presence of a 6.9 Mb mosaic 22q11 deletion (approximately 50% of cells) in peripheral blood. These rearrangements not only changed the order, but also the amount of genetic material. Analysis through DECIPHER databases the 6.9M deletion could lead to two syndromes: 22q11 deletion syndrome (the phenotypes are abnormality of the heart, delayed speech and language development, hypocalcemia, nasal speech, and T lymphocytopenia) and 22q11.2 distal deletion syndrome (the phenotypes are abnormality of the face, intellectual disability, short stature, and small for gestational age). Additionally, symptoms such as Tetralogy of Fallot (Chen et al., 2004), hypoplastic left heart syndrome (Consevage et al., 1996), and congenital anomalies have been previously reported associated with mosaic 22q11.2 deletion. It was unexpected that the patient did not show any of these symptoms. The clinical manifestations of this patient were merely oligoasthenozoospermia, mild bradycardia, and mild tricuspid regurgitation, suggesting that the results of peripheral blood cytogenetic testing were not consistent with clinical symptoms.

The patient complained that he had not been exposed to radioactive source and other substances that may cause chromosomes aberrations. We assumed that this patient might be a mosaic individual. The generation of genetically distinct cells from a single zygote necessitates postzygotic de novo mutational events (Veltman & Brunner, 2012) as the cause of mosaicism. There are some specific types of mosaicism that describe which parts of the body harbor the variant cells and the potential for transmission to offspring. These include germ line mosaic (also known as gonadal mosaicism), somatic mosaicism, and gonosomal mosaicism (a combination of germ line and somatic mosaicism) (Biesecker & Spinner, 2013). At the level of the whole organism, appreciation of the mosaic phenotype depends on tissue‐to‐tissue genetic variations (Youssoufian & Pyeritz, 2002).

It was reported that some mosaic disorders are caused by mutations that were seen only in mosaic form and that were incapable of germ line transmission (Happle, 1986, 1987). This might be caused by different mechanisms, for example, although a somatic mutation might occur in any cell, if this cell was a gonadal precursor cell and the mutation specifically caused apoptosis in the germ cells. Meiotic segregation studies in males carrying a reciprocal translocation showed the proportion of chromosomally unbalanced spermatozoa to vary from 10 to >80% (Benet, Oliver‐Bonet, Cifuentes, Templado, & Navarro, 2005; Morel et al., 2004).

The main purpose of this patient was to consult the cause of infertility, so we tested sperm DNA by CNV‐seq analysis to evaluate the genetic material of gametes. The result showed that the rate of 22q11 deletion sperms was obviously lower than peripheral blood cells; the frequency of gametes exhibiting a normal or balance chromosomal equipment was above 80% in sperm samples. Fortunately, this patient decided to try an IVF with microinjection and had a chance to get a normal offspring.

To the best of our knowledge, our report is the first case of a de novo gonosomal mosaic of chromosome 22q11 deletion associated with male infertility, meanwhile, without other severe clinical manifestations.

## CONFLICT OF INTEREST

None.

## AUTHOR CONTRIBUTION

H. Z. performed the FISH assay; T. H. and Z. Z. performed the CMA assay; J. W. performed the CNV‐seq assay; X. Z., Y. L., J. Z., D. X., B. X., L. Q., and L.X. performed the karyotype assay; Y. L. and H.S. analyzed the data; S. L. and H.W. coordinated the project. Y.L. and H.S. wrote the paper.
